# Novel Schiff Bases Based on the Quinolinone Skeleton: Syntheses, X-ray Structures and Fluorescent Properties

**DOI:** 10.3390/molecules190913509

**Published:** 2014-09-01

**Authors:** Zdeněk Trávníček, Roman Buchtík, Ivan Nemec

**Affiliations:** Regional Centre of Advanced Technologies and Materials, Department of Inorganic Chemistry, Faculty of Science, Palacký University, 17 listopadu 12, CZ-77146 Olomouc, Czech Republic; E-Mails: roman.buchtik@upol.cz (R.B.); ivan.nemec@upol.cz (I.N.)

**Keywords:** 2-phenyl-3-amino-4(1*H*)-quinolinone, Schiff base, fluorescence, *in vitro* cytotoxicity, X-ray structures

## Abstract

A series of a new type of Schiff bases **1**–**7**, derived from 2-phenyl-3-amino-4(1*H*)-quinolinone and R-salicyladehyde derivatives wherein R = 3-hydroxy (**1**), 3,4-dihydroxy (**2**), 3-methoxy (**3**), 3-carboxy (**4**), 3-allyl (**5**), 5-chloro (**6**), and 5-nitro (**7**), was synthesized and structurally characterized. Each of the molecules **1**, **3** and **7** consists of three planar moieties (*i.e*., a quinolinone and two phenyl rings), which are mutually oriented differently depending on the appropriate substituent R and the extent of non-covalent contacts stabilizing the crystal structures. The compounds were studied for their fluorescence properties, where compound **6** yielded the strongest intensity both in the solid phase and in 100 μM ethanol solution with a quantum yield of φ = 3.6% as compared to quinine sulfate used as a standard. The *in vitro* cytotoxicity of these compounds was tested against the human osteosarcoma (HOS) and breast adenocarcinoma (MCF7) cell lines, revealing no activity up to the concentration of 50 µM.

## 1. Introduction

**Scheme 1:**
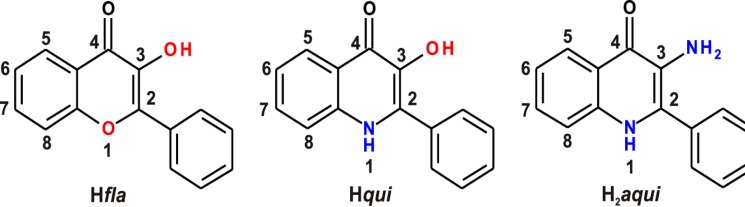
Schematic representations of 3-hydroxyflavone (*left*), 2-phenyl-3-hydroxy-4(1*H*)-quinolinone, H*qui*, (*middle*) and 2-phenyl-3-amino-4(1*H*)-quinolinone, H_2_*aqui*, (*right*) showing their structural similarity.

The very first paper reporting the synthesis of 2-phenyl-3-amino-4(1*H*)-quinolinones dates back to 1966 [1], nevertheless, a novel synthetic approach described in 2006 made these compounds more easily available [2]. 2-Phenyl-3-amino-4(1*H*)-quinolinone (H_2_*aqui*) and its derivatives represent a group of 4-quinolones isosteric with 3-hydroxyflavones (H*fla*) (differing structurally in the aromatic heteroatom in the position 1, *i.e*., the oxygen in flavones is substituted by nitrogen in H_2_*aqui* (Scheme 1), which are natural plant pigments exhibiting antioxidant [3] and anti-inflammatory properties, [4] and also with 2-phenyl-3-hydroxy-4(1*H*)-quinolinones (H*qui*) (the structural difference then being within the aromatic ring in position 3, *i.e*., the hydroxyl group in H*qui* is substituted by an amino group in H_2_*aqui* (Scheme 1). No biological activity has been found any in non-substituted H*qui*, although many of its derivatives exhibit cytotoxicity via a topoisomerase inhibition mechanism [5,6,7]. To date, remarkable *in vitro* cytotoxicity results have been obtained for the compounds carrying 3,5-dichloro-4-amino substituents on the 2-phenyl ring, which is currently the only known structural pattern positively affecting the cytotoxic activity [8] (with the best IC_50_ = 0.25–0.56 μM for 2-(4-amino-3,5-dichlorophenyl)-3-hydroxy-4(1*H*)-quinolinone-7-dodecylcarboxamide) against CEM (human T cell lymphoblast-like cells), K562 (human myelogeneous leukemia) and HCT116 (human colon cancer cells). On the other hand, as published previously [9,10,11], our group managed to use the originally inactive H*qui* as a ligand for the preparation of some highly cytotoxic Cu(II) complexes (with IC_50_ = 0.36–0.56 μM for [Cu(*qui*)(mphen)]BF_4_·H_2_O, where mphen = 5-methyl-1,10-phenanthroline), against A2780 ovarian and A2780cis *cisplatin*-resistant ovarian carcinomas.

In this work, we tried to find out whether and/or how a change in the substitution in the position 3 of the quinolinone skeleton affects the *in vitro* cytotoxicity against chosen types of cancer cell lines. In other words, we sought to find another possible skeletal or building pattern that would positively influence the cytotoxic effects. Nevertheless, biological activity is not the only reason why we focused on these 4-quinolinones. These compounds are also characteristic for their intrinsic photoluminescence [12]. Photoluminescence effects, such as fluorescence, could be utilized as a tool in the study of cytotoxic compounds. Recently, it has been also found that variously substituted H*qui* compounds might serve as efficient fluorescent labels for the observation of biological systems or processes [13,14].

Herein, we focused on the preparation of a series of novel Schiff bases based on the condensation of non-substituted H_2_*aqui* with variously substituted salicylaldehydes. Such synthetic objective was chosen due to the simple preparation of the Schiff bases via nucleophilic addition of the 3-amino group of quinolinone to the aldehyde group of the corresponding aromatic 2-hydroxyaldehyde.

## 2. Results and Discussion

### 2.1. Synthesis

**Scheme 2:**
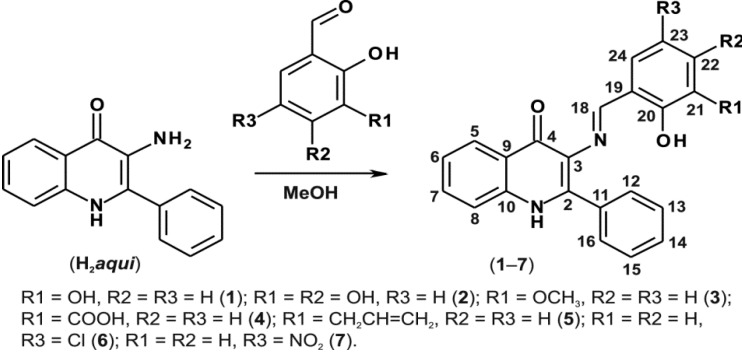
A schematic representation of the synthesis of compounds **1**–**7** with the corresponding ^1^H- and ^13^C-NMR numbering.

In order to prepare the Schiff bases **1**–**7**, we used H_2_*aqui* synthesized according to the previously reported procedure [2], and performed a reaction with the corresponding commercially available salicylaldehydes as illustrated in Scheme 2. The mixtures were refluxed in methanol and the products were isolated in relatively good yields (65%–89%).

### 2.2. General Characterizations

The chemical purity and composition of the prepared compounds **1**–**7** were checked by a combination of different techniques, *i.e*., elemental analysis, FTIR and multinuclear NMR spectroscopy, and mass spectrometry. After several efforts to prepare single crystals suitable for X-ray analysis, we were able to obtain the crystals for compounds **1** and **7** by slow evaporation of the mother liquors or recrystallization from methanol in the case of **3**. The colour of the prepared compounds varied from bright yellow through orange to red in the case of carboxyl derivative **4**. All the compounds are very soluble in DMF and DMSO.

There are several characteristic vibration bands in the FTIR spectra. The first is a sharp peak of a medium intensity found in the 3,275–3,250 cm^–1^ range. It may be assigned to the stretching *v*(N–H) vibration of the aromatic amine group of the quinolinone skeleton. Valence vibrations of aromatic C–H groups may be found in the 3,050–3,150 cm^−1^ region. Another characteristic vibration was found in the 1,626–1,623 cm^–1^ region which can be attributed to the ν(C=O) vibration of the quinolinone moiety. Adjacent peaks in the 1,610–1,600, 1,579–1,557 cm^–1^ and 1,476–1,462 cm^–1^ regions could be assigned to ν(C^…^N) or ν(C^…^C). The stretching vibrations of the hydroxyl moieties cannot be easily identified because of the expectable red shift and broadening of the band peaks due to the formation of the relatively strong O–H···O, N–H···O and O–H···N hydrogen bonds. Compound **2** shows an additional OH– group band at 3536 cm^−1^, which probably originates from the presence of methanol molecules in the crystal lattice of this compound. Vibrational bands of the different peripheral groups on the salicylaldiminato moiety can be identified by mutual comparison of the spectra: the carboxylic ν(C=O) band at 1684 cm^–1^ in **4**; the characteristic R–NO_2_ group strong bands at 1528 and 1334 cm^–1^ in **7**, the ν(O–CH_3_) vibrations in **3** at 1243 and 1223 cm^–1^; and the allyl group in **5** exhibits its aliphatic stretching ν(C=C) vibration at 903 cm^–1^ [15,16].

In the ^1^H-NMR spectra, the chemical shifts of the O–H salicylic group appear in the region of δ 12.53–12.89 ppm, except for **4** and **7**, where it appears at δ 14.89 and δ 13.94 ppm, respectively. Such a difference in the shift is caused by the adjacent electron withdrawing groups, *i.e*., COOH in **4** and R–NO_2_ in **7**. On the other hand, the H–N of the quinolinone moiety was not so strongly affected by the different substituents of the salicylaldiminato ring and thus the chemical shifts were observed in a shorter range varying from δ 12.09 ppm (compound **2**) to δ 12.66 ppm (compound **4**). The ^13^C APT NMR experiments showed correct numbers and intensities of signals as can be seen in the representative spectrum of **3** depicted in Supplementary Materials Figure S1. Concretely in this spectrum the non-aromatic carbon signal at δ 55.91 ppm may be assigned to the methoxy group. The signal at δ 172.14 ppm in compound **4** belongs to the carboxylate carbon. The carbon atoms of the allyl group in compound **5** were found at δ 33.32 (the terminal methylene carbon at position 27), 116.17 (methylene carbon at position 29 bound to phenyl ring) and 136.90 ppm (the methine group carbon at position 28). Assignments of the hydrogen and carbon atoms of the quinolinone and phenyl rings were carried out using 2D correlation experiments ^1^H−^1^H gs-COSY, ^1^H−^13^C gs-HMQC and ^1^H−^13^C gs-HMBC (see Section 3.2. and Supplementary Materials Figure S2).

Electrospray-ionization (ESI) mass spectrometry revealed that the molecular peaks were found in the spectra of all the studied compounds **1**–**7** measured in the 50-2000 m/z range. The ESI+ mode detected [M+H]^+^ as the most abundant molecular peak for **1**, **2**, **5** and **6**. This positive-mode ESI mass spectra also detected dimers of molecular peaks of these compounds [2M+H]^+^ of minor intensity (20%–25%). In the case of **7** the most abundant peak was the dimer, while the molecular peak was detected at abundance of 85%. For the characterization of compounds **3** and **4**, the negative-mode ESI was used due to a weak detectability of the molecular peaks in the positive mode. ESI− detected [M−H]^−^ molecular peaks together with minor peaks of the dimers [2M−H]^−^ (Supplementary Materials Figure S3).

### 2.3. X-ray Structure Characterizations

**Figure 1:**
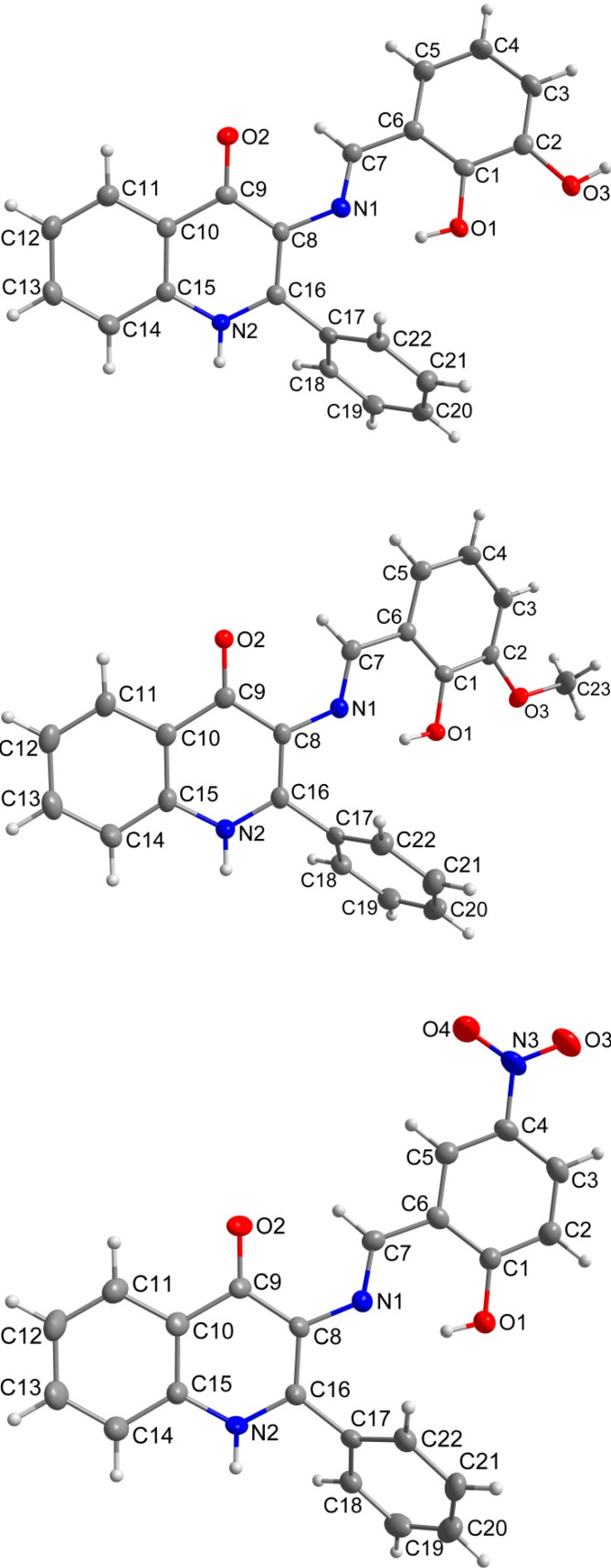
Molecular structures of compounds **1** (top), **3** (middle) and **7** (bottom) together with the atom numbering scheme. The water molecule of crystallization in compound **1** was omitted for clarity. The non-H atoms are drawn as thermal ellipsoids at the 50% probability level.

The crystals suitable for the single-crystal X-ray diffraction experiments were successfully prepared for **1**, **3** and **7**. The crystal data and structure refinements are given in Table 1. The molecular structures of the Schiff base molecules are very similar in all three cases (Figure 1). The molecules consist of a planar quinolinone moiety, which is substituted by a phenyl ring in the position 2 and by the salicylaldimino moiety in the position 3. The salicylaldimino moiety is variously substituted (besides the hydroxyl group in the position 2: 3–OH in **1**, 3–OCH_3_ in **3** and 5–NO_2_ in **7**) and it holds the dihedral angle to the quinolinone ring (when considering the angle of the least square planes of the aromatic moieties) of 21.75(5)°, (**1**), 21.64(4)°, (**3**), 6.04(8)°, (**7**)). The phenyl ring in the position 2 forms a dihedral angle of 56.9(4)° (for **1**), 65.1(5)° (for **3**), 54.0(5)° (for **7**) with the quinolinone skeleton. Mutual orientation between the salicylaldimino and quinolinone rings seems to be influenced by the relatively strong intramolecular O–H···N hydrogen bond between the oxygen atom from the *ortho*-hydroxyl group (O_OH_) and nitrogen atom from the imino group, where a shorter hydrogen bond implies a wider dihedral angle: *d*(O1···N1) = 2.599(2) Å, (**1**), 2.565(2) Å, (**3**), 2.616(3) Å, (**7**).

**Table 1:** Crystal data and structure refinements for compounds (**1**), (**3**) and (**7**).

	(1)	(3)	(7)
Formula	C_22_ H_18_ N_2_ O_4_	C_23_ H_18_ N_2_ O_3_	C_22_ H_15_ N_3_ O_4_
M (g mol^−1^)	374.38	370.39	385.38
*T* (K)	100(2)	100(2)	120(2)
Crystal system, space group	Monoclinic, *P*2_1_/*c*	Orthorhombic, *P*2_1_2_1_2_1_	Orthorhombic, *Fdd*2
*Unit cell dimensions*			
*a* (Å)	10.5110(4)	9.4567(4)	12.6476(10)
*b* (Å)	8.4997(3)	11.5154(6)	23.833(4)
*c* (Å)	22.1971(8)	16.8507(7)	23.837(3)
*α* (°)	90	90	90
*β* (°)	102.487(4)	90	90
*γ* (°)	90	90	90
*V* (A^3^)	1934.62(12)	1835.00(14)	7185.2(16)
*Z, D_c_* (g cm^−3^)	4, 1.285	4, 1.341	16, 1.425
*F* (000)	784	776	3199
*θ* range for data collection (°)	3.02 ≤ θ ≤ 25.00	3.00 ≤ θ ≤ 24.99	3.15 ≤ θ ≤ 25.00
Reflections collected/unique	17,842/3400(0.0324)	12,396/3225(0.0425)	15,283/2946(0.0952)
Data/restraints/parameters	3400/3/261	3225/0/255	2946/1/263
Goodness-of-fit on *F^2^*	1.065	0.975	0.948
Final *R* indices [*I* > 2σ(*I*)]	0.0393, 0.1104	0.0322, 0.0702	0.0431, 0.0975
*R* indices (all data)	0.0574, 0.1152	0.0418, 0.0723	0.0593, 0.1020
Largest peak and hole (e Å^−3^)	0.306, −0.406	0.152, −0.194	0.189, −0.227
CCDC Number	1008032	1008033	1008034

As can be expected, the C−O bond lengths in the OH groups (in Å, 1.352(2) and 1.369(2), (**1**); 1.348(2), (**3**); 1.333(3), (**7**)) or in the methoxy groups (1.376(2) Å, (**3**)) are longer than the C=O lengths in the keto groups (in Å, 1.265(2), (**1**); 1.244(2), (**3**); 1.254(3), (**7**)). Similarly, the imino C=N bond lengths are shorter (in Å, 1.284(2), (**1**); 1.296(2), (**3**); 1.279(3), (**7**)), than the C^…^N bonds in the quinolinone ring (in Å, 1.345(2) and 1.364(2), (**1**); 1.348(2) and 1.382(2), (**3**); 1.353(3) and 1.377(3), (**7**)).

The crystal structures of the title compounds are rich in the intermolecular non-covalent interactions due to the presence of several groups available for their formation. For the overview of the non-covalent interactions see Supplementary Materials Table S1.

In compounds **1** and **3**, the preference of the quinolinone N–H group for the bifurcated hydrogen bonding with two *ortho*-located oxygen atoms from the imino part can be observed. In this supramolecular assembly, the bulkier 3-methoxy group in (**3**), which should be a weaker acceptor of the hydrogen bonding than the hydroxyl group, provides N−H···O hydrogen bond of the almost same donor···acceptor (D···A) distance as the 3-hydroxyl acceptor in **1**: *d*(N2···O3) = 2.885(2) Å, (**1**), 2.871(2) Å, (**3**). On the other hand, the significant length difference is observed in the complementary N−H···O hydrogen bond with the 2-hydroxyl acceptor: *d*(N2···O1) = 3.067(2) Å, (**1**), 3.198(2) Å, (**3**). The crystal structure of these two compounds is also stabilized by the offset *π−π* stacking between the phenyl ring from the imino part and the quinolinone 2-phenyl ring of the neighbouring molecules with the centroid-centroid distances of 3.805(2) Å in **1** and 3.748(2) Å in **3**.

**Figure 2:**
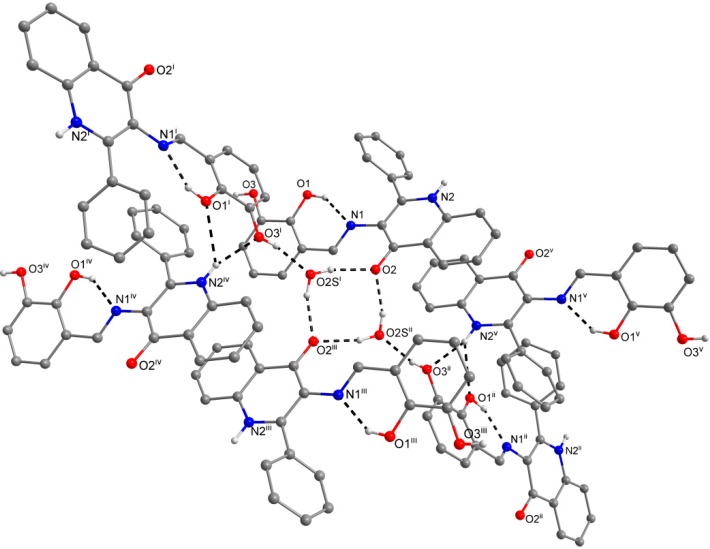
A part of the crystal structure of (**1**) showing the N−H···O, O–H···Oand O−H···N hydrogen bonds (black dashed lines). The H–atoms not involved in the interactions are omitted for clarity. Symmetry codes: (i) − x, − 0.5 + y, 0.5 + z; (ii) x, 1.5 − y, − 0.5 + z; (iii) – x, 1 − y, − z; (iv) − 1 + x, y, z; (v) 1 − x, 1 − y, − z.

**Figure 3:**
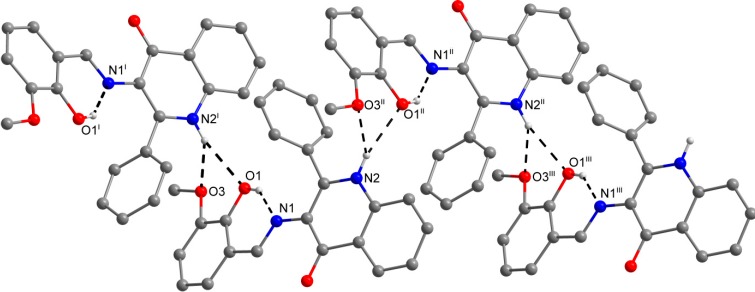
A part of the crystal structure of (**3**) showing the N−H···O and O−H···N hydrogen bonds (black dashed lines). The H–atoms not involved in the interactions are omitted for clarity. Symmetry codes: (i) 1 – x, –0.5 + y, 0.5 – z; (ii) 1 – x, 0.5 + y, 0.5 – z; (iii) x, 1 + y, z.

**Figure 4:**
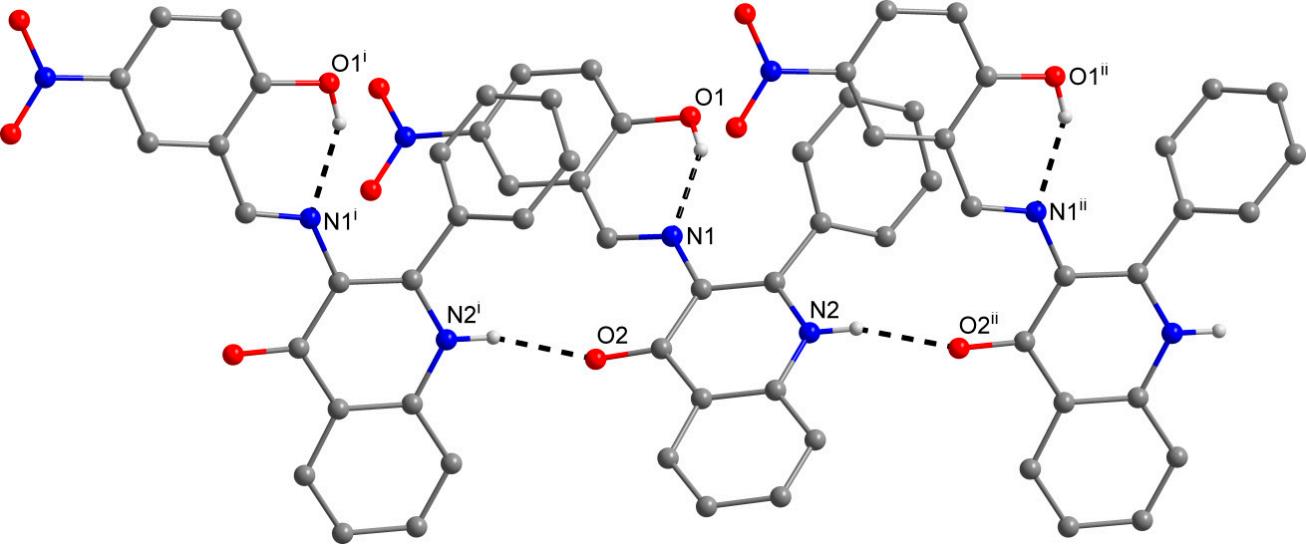
A part of the crystal structure of (**7**) showing the O−H···N and N−H···O hydrogen bonds (black dashed lines). The H–atoms not involved in the interactions are omitted for clarity. Symmetry codes: (i) 0.25 + x, 0.25 – y, 0.25 + z; (ii) –0.25 + x, 0.25 – y, –0.25 + z.

The role of the keto-oxygen atoms differs between the **1** and **3** and it underlines different structural properties arising from different R1 substituents (according to the Scheme 2) of the imino moiety. In **1** the keto-oxygen atom is involved in the centrosymmetric supramolecular synthon supported by the water molecules in a bridging function (Figure 2). The interconnection between adjacent molecules is provided by relatively strong hydrogen bonding: *d*(O2s···O2) = 2.711(2) and 2.751(2) Å. The water molecule acts also as a hydrogen bonding acceptor and it is stabilized by a very short interaction with the 3-hydroxyl group: *d*(O3···O2s) = 2.595(2) Å. In summary, all the above mentioned non-covalent interactions expand the structure of (1) into a 3D network. In **3**, the keto oxygen atom accepts only the weak C–H···O interactions with the shortest contact arising from the methoxy group (*d*(C8···O2) = 3.270(2) Å) (Figure 3). In **7** the keto oxygen atom is involved in the formation of the chain substructure composed of adjacent molecules interconnected by hydrogen bonding between the quinolinone N−H group and keto oxygen atom: *d*(N2···O2) = 2.806(3) Å (Figure 4). The chain-like alignment is supported by the C–H···O contact with *d*(C7···O2) = 3.348(3) Å. Another important C–H···O contact is found between the C−H group located on the quinolinone 2-phenyl ring and the adjacent oxygen atom from the nitro group: *d*(C13···O4) = 3.315(4) Å. From the other non-covalent contacts found in **7** the close intermolecular and centrosymmetric N···O contact between neighbouring nitro groups with *d*(N3···O3) = 3.043(3) Å, which is shorter than the sum of their van der Waals radii (3.25 Å) is worth mentioning. The above mentioned (for **1** and **3**) offset *π−π* stacking between the quinolinone 2-phenyl ring and the phenyl ring of the imino part is too distant with the centroid-centroid distance equal to 4.1783(6) Å, but the other ring-ring interactions between the imino and quinolinone ring moieties can be found with the centroid-centroid distance equal to 3.4330(4) Å.

### 2.4. Fluorescence Spectroscopy Studies

**Figure 5:**
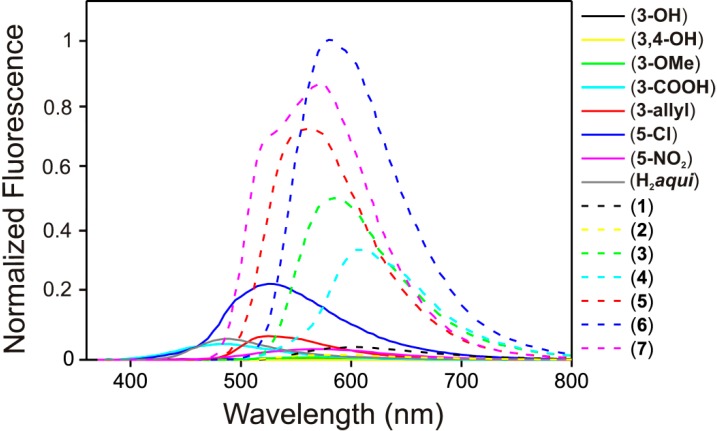
Normalized solid state fluorescence spectra of compounds **1**–**7** and starting compounds (salicylaldehydes and H_2_*aqui*). The comparison of intensities for the Schiff bases **1**–**7** and corresponding starting compounds, where (**3**–**OH**) = 3-hydroxy-salicylaldehyde, (**3**,**4**–**OH**) = 3,4-dihydroxysalicylaldehyde, (**3**–**OMe**) = 3-methoxy-salicylaldehyde, (**3**–**COOH**) = 3-carboxy salicylic acid, (**3**–**allyl**) = 3-allylsalicylaldehyde, (**5**–**Cl**) = 5-chlorosalicylaldehyde, (**5**–**NO_2_**) = 5-nitrosalicylaldehyde and (H_2_*aqui*) = 2-phenyl-3-amino-4(1*H*)-quinolinone.

Due to the fact that 4-quinolinones often exhibit fluorescence, we sought to find out whether and/or how the additional aromatic salicylic ring, forming thus a Schiff base, can affect this property. Firstly, we measured the UV–Vis spectra of the compounds in EtOH–H_2_O (95:5 v/v) and in the solid state in order to find out the absorption maxima (see Supplementary Materials Figure S4−S5). The solid state absorption maxima were found in the region between 370 and 440 nm and thus we decided to use the fluorescence excitation wavelength at 400 nm as an approximately mean value. Solid state fluorescence measurements were carried out for the starting compounds (corresponding salicylaldehydes and H_2_*aqui*) as well as the compounds **1**–**7**. Overall, the compounds **1**–**7** exhibited significantly higher fluorescence in comparison with the starting compounds (Figure 5). Compounds **1** and **2** yielded the weakest fluorescence comparable to the rest of the starting salicylaldehydes and thus also to the compounds **3**–**7**. This is most probably caused by the quenching process decreasing the intensity of fluorescence due to the rich intermolecular hydrogen bonding caused by peripheral hydroxyl groups [17]. If the hydroxyl groups are substituted by methoxy or carboxylic groups, the relative intensity significantly rises as can be seen in the case of compounds **3** and **4**. However, the most significant rise in the relative intensity was observed for compounds **5**, **6** and **7**, which do not have any peripheral groups appropriate for hydrogen bonding on the imino moiety. Dual fluorescence, otherwise characteristic for derivatives of H*qui* [13] has been observed only in the case of **7** with two partly overlapped bands. After performing of the Gauss deconvolution of the spectrum, two fluorescence intensity maxima have been identified at 521 nm and 575 nm (see Supplementary Materials Figure S6).

**Figure 6:**
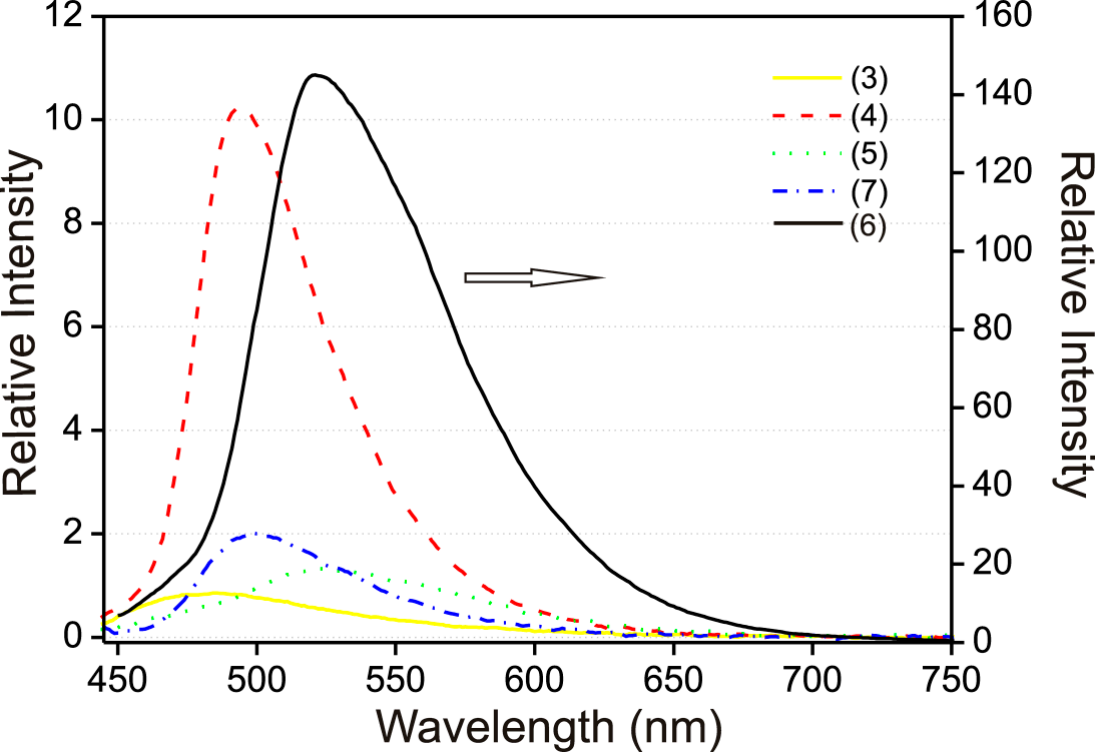
Fluorescence emission spectra of compounds (**3**–**5**, **7**) (*left y-axis*) and (**6**) (*right y-axis*) 100 μM in EtOH–H_2_O (95:5 v/v). Excitation wavelength was fixed at the absorption maximum of each compound.

The 100 μM solution spectra in EtOH–H_2_O (95:5 v/v) were obtained only for compounds **3**–**7**, due to the limited solubility of (**1**) and (**2**) in the solvent mixture. The emission maxima in the solution spectra compared to the solid phase were in all cases shifted to lower wavelengths (blue-shifted) and also significant decrease in the fluorescence intensity in the EtOH–H_2_O (95:5 v/v) solution was observed (Figure 6). While the solid state emission maximum for compound (**3**) λ_max_ = 585 nm, in solution λ_max_ = 485 nm, in the case of compound (**4**) solid λ_max_ = 605 nm, in solution λ_max_ = 495 nm, for compound (**5**) solid λ_max_ = 560 nm, solution λ_max_ = 520 nm, for compound (**6**) solid λ_max_ = 580 nm, solution λ_max_ = 500 nm and for compound (**7**) solid λ_max_ = 575 nm, solution λ_max_ = 520 nm (see Table 1).

The quantum yields calculated from fluorescence intensities compared to the standard solution of quinine sulfate were relatively low, for compound (**3**) it was 0.6% of the quinine intensity. Compounds (**4**–**7**) exhibited slightly higher intensity with results listed in Table 2. Unlike the solid spectra stated above, the EtOH–H_2_O (95:5 v/v) solution spectrum of (**7**) exhibited only one fluorescence maximum (see Figure 6). 

**Table 2:** Comparison of fluorescence emission maxima in the solid state and EtOH–H_2_O (95:5 v/v) for compounds (**3**–**7**) together with calculated quantum yields in the EtOH–H_2_O (95:5 v/v) solution compared to standard quinine sulfate in 0.1 M sulfuric acid.

Compound	Solid λ_max_ (nm)	EtOH–H_2_O (95:5 v/v) λ_max _(nm)	Quantum Yield (%) *
3	585	485	0.6
4	605	495	3.4
5	560	520	2.5
6	580	500	3.6
7	575	520	1.3

*: compared to standard quinine sulfate in 0.1 M sulfuric acid (φ = 0.54).

### 2.5. In Vitro Cytotoxicity

A noticeable structural similarity of compounds **1**–**7** with biologically active cytotoxic 2-phenyl-3-hydroxy-4(1*H*)-quinolinones led us to the determine whether these compounds show any *in vitro* cytotoxic activity or not. In order to find this out, we carried out *in vitro* cytotoxicity screening of compounds **1**–**7** together with H_2_*aqui* and *cisplatin* (used as a standard) against two human cancer cell lines (human osteosarcoma HOS and human breast adenocarcinoma MCF7). The MTT assay was used to study *in vitro* cytotoxicity of **1**–**7** only up to the concentration of 50.0 μM due to the generally accepted fact, that testing to the higher concentration limit is not meaningful from the biological/therapeutic point of view and also in connection with a limited solubility of the tested compounds in the testing media used (0.1% DMF in distilled water). Unfortunately, the obtained results showed the compounds **1**–**7**, as well as H_2_*aqui*, as being inactive within the studied concentration ranges (*i.e*., with IC50 > 50.0 μM). The reference standard, *cisplatin*, showed moderate *in vitro* cytotoxicity with IC50 = 25.4 ± 8.5 μM against HOS and 18.1 ± 5.1 μM against MCF7.

## 3. Experimental Section

### 3.1. General Methods

Elemental analysis (C, H, N) was carried out on a Flash EA‑2000 Elemental Analyser (Thermo Scientific, Waltham, MA, USA). FTIR spectra were recorded on a Nexus 670 FTIR (Thermo Nicolet, Waltham, MA, USA) using the ATR technique in the range of 400–4000 cm^–1^. The intensities of reported FTIR signals are defined as s = strong, m = medium, w = weak and vs = very strong. Mass spectra (MS) were recorded on an LTQ Fleet (Thermo Scientific, QualBrowser software, version 2.0.7, Thermo Fischer Scientific) spectrometer using the positive (ESI+) and negative (ESI−) electrospray ionisation and full scan mode in the 10 μM methanol or acetonitrile solutions. Melting points were measured on a Büchi MeltingPoint B-545 apparatus and were uncorrected. ^1^H and ^13^C NMR spectra and 2D correlation experiments (^1^H–^1^H gs-COSY, ^1^H–^13^C gs-HMQC and ^1^H–^13^C gs-HMBC; gs = gradient selected, COSY = correlation spectroscopy, HMQC = heteronuclear multiple quantum coherence and HMBC = heteronuclear multiple bond coherence) of *d_6_*-DMSO solutions were carried out at 300K on a Varian 400 MHz spectrometer (Santa Clara, CA, USA) at 400.00 MHz (^1^H) and 100.58 MHz (^13^C). The spectra were adjusted against the signals of tetramethylsilane (TMS). The splitting of proton resonances in the reported ^1^H spectra is defined as *s* = singlet, *d* = doublet, *dd* = doublet of doublets, *t* = triplet, *tt* = triplet of triplets, *br* = broad band, *m* = multiplet.

### 3.2. Materials

Chemicals and solvents were purchased from Sigma-Aldrich Co. (Prague, Czech Republic) and Acros Organics Co. (Pardubice, Czech Republic), and were used as received. 2-Phenyl-3-amino-4(1*H*)-quinolinone was synthesized according to the previously reported procedure [2] and characterized by elemental analysis and ESI+ mass spectrometry (see Supplementary Materials).

### 3.3. Synthesis: General preparation of Compounds **1**–**7**

2-Phenyl-3-amino-4(1*H*)-quinolinone (0.5 g, 2.12 mmol) was dissolved in methanol (40 mL) and the corresponding aldehyde (2.5 mmol) in methanol (5 mL) was added. After stirring for 10 min, the reaction mixture was slowly heated to reflux and refluxed for 2–3 h. During this time, a crystalline precipitate began to form. After cooling down the reaction mixture, the solid formed was filtered off and washed with cold methanol. The products were dried under vacuum. Yields: 65%–89%. Based on the results of the techniques used (see below), the compounds were prepared in sufficient purity and no further purification was needed except for **4**, which was recrystallized from acetic acid.

*2-Phenyl-3-[N-(2,3-dihydroxybenzylidenamino)]-4(1H)-quinolinone·H_2_O* (**1**). Bright orange crystalline product; yield: 523 mg (66%), mp 220 °C; Anal. Calc. for C_22_H_18_N_2_O_4_ (M_r_ = 374.4): C, 70.6; H, 4.9; N, 7.5. Found: C, 70.4; H, 5.0; N, 7.3%; ESI+ *m/z* (Int. %): 357 [M+H]^+^ (100), 713 [2M+H]^+^ (20); ^1^H-NMR, DMSO*-d*_6_, *δ* (ppm), *J* (Hz): 6.69, 1H, *t*, 7.7, HC^23^, 6.81, 1H, *d*, 7.7, HC^22^, 6.88, 1H, *d*, 7.7, HC^24^, 7.39, 1H, *t*, 6.9, HC^6^, 7.58, 3H, *m*, HC^13–15^, 7.63, 2H, *m*, HC^12,16^, 7.70, 2H, *m*, HC^7,8^, 8.25, 1H, *d*, 8.2, HC^5^, 8.88, 1H, *br*, HO^21'^ 10.04, *s*, HC^18^, 12.20, *br*, HN^1^, 12.53, 1H, *s*, HO^20'^; ^13^C-NMR, DMSO*-d*_6_, *δ* (ppm): 118.47 (C8), 118.90 (C22), 119.10 (C6), 120.56 (C19), 122.82 (C23), 124.05 (C24), 124.53 (C9), 125.92 (C5), 126.10 (C11), 129.02 (C13,15), 129.90 (C12,16), 130.36 (C14), 132.26 (C7), 134.10 (C2), 138.60 (C3), 145.70 (C10), 147.94 (C21), 149.01 (C20), 164.66 (C18), 172.79 (C4); FTIR (ν, cm^–1^): 3387vs, 3275vs, 3220br, 3160br, 3114br, 3062br, 3000br, 1626s, 1608s, 1567vs, 1524s, 1503s, 1470vs, 1453s, 1388s, 1348vs, 1264s, 1241s, 1191s, 1174s, 1072m, 982m, 920m, 840m, 755s, 734s, 700s, 670m, 585w, 515w, 493w, 470w.

*2-Phenyl-3-[N-(2,3,4-trihydroxybenzylidenamino)]-4(1H)-quinolinone* (**2**). Orange crystalline product; yield: 508 mg (65%), mp 290 °C; Anal. Calc. for C_22_H_16_N_2_O_4_ (M_r_ = 372.4): C, 71.0; H, 4.3; N, 7.5. Found: C, 70.8; H, 4.3; N, 7.4%; ESI+ *m/z* (Int. %): 373 [M+H]^+^ (100), 745[2M+H]^+^ (25); ^1^H-NMR, DMSO*-d*_6_, *δ* (ppm), *J* (Hz): 6.36, 1H, *d*, 8.5, HC^23^, 6.76, 1H, *d*, 8.5, HC^24^, 7.36, 1H, *t*, 7.2, HC^6^, 7.56, 3H, *m*, HC^13-15^, 7.63, 3H, *m*, HC^7,12,16^, 7.69, 1H, *m*, HC^8^, 8.20, 1H, *br*, HC^18^, 8.23, 1H, *d*, 7.9, HC^5^, 9.48, 1H, *s*, HO^22'^, 9.81, 1H, *s*, HO^21'^, 12.09, 1H, *s*, HN^1^ , 12.87, 1H, *s*, HO^20'^; ^13^C-NMR, DMSO*-d*_6_, *δ* (ppm): 107.70 (C23), 113.35 (C19), 119.02 (C8), 123.81 (C6), 123.83 (C14), 125.07 (C11), 125.87 (C9), 125.90 (C5), 128.97 (C13,15), 129.94 (C12,16), 130.24 (C24), 132.07 (C7), 132.59 (C21), 134.25 (C2), 138.61 (C3), 146.62 (C20), 149.91 (C10), 150.85 (C22), 166.07 (C18), 172.71 (C4); FTIR (ν, cm^–1^): 3536s, 3264br, 3224br, 3104br, 3066br, 3001br, 1625vs, 1611vs, 1570vs, 1476vs, 1447vs, 1367s, 1332vs, 1277vs, 1264vs, 1226vs, 1195vs, 1162vs, 1120s, 1078vs, 1027m, 979s, 935m, 917m, 853m, 786m, 756s, 695s, 647m, 588m, 538w, 516w, 489w, 459w.

*2-Phenyl-3-[N-(2-hydroxy-3-methoxybenzylidenamino)]-4(1H)-quinolinone* (**3**). Bright orange crystalline product; yield: 621 mg (79%), mp 271 °C; Anal. Calc. for C_23_H_18_N_2_O_3_ (M_r_ = 370.4): C, 74.6; H, 4.9; N, 7.6. Found: C, 74.5; H, 4.9; N, 7.5%; ESI− *m/z* (Int. %): 369 [M–H]^–^ (100), 739[2M–H]^–^ (10). Crystals suitable for single crystal X-ray analysis were obtained by re-crystallization of the compound from methanol; ^1^H-NMR, DMSO*-d*_6_, *δ* (ppm), *J* (Hz): 3.69, 3H, *s*, HC^27^, 6.79, 1H, *t*, 8.1, HC^23^, 6.95, 1H, *d*, 8.1, HC^22^, 7.00, 1H, *d*, 8.1, HC^24^, 7.39, 1H, *t*, 7.4, HC^6^, 7.59, 3H, *m*, HC^13–15^, 7.65, 2H, *m*, HC^12,16^, 7.70, 2H, *m*, HC^7,8^, 8.25, 1H, *d*, 9.2, HC^5^, 10.17, *s*, HC^18^, 12.25, *br*, HN^1^, 12.65, 1H, *s*, HO; ^13^C-NMR, DMSO*-d*_6_, *δ* (ppm): 55.87 (C27), 114.70 (C24), 118.66 (C8), 119.09 (C5), 120.31 (C19), 123.94 (C14), 124.06 (C6), 124.15 (C9), 125.87 (C23), 126.25 (C11), 129.02 (C13,15), 129.77 (C12,16), 130.31 (C7), 132.28 (C22), 134.10 (C21), 138.60 (C2), 148.03 (C3), 148.52 (C10), 150.19 (C20), 163.79 (C18), 173.03 (C4); FTIR (ν, cm^–1^): 3273s, 3231s, 3176s, 3056m, 3004m, 2967m, 2939m, 2841m, 1624s, 1611s, 1577vs, 1510s, 1462vs, 1436vs, 1389s, 1346s, 1241vs, 1188s, 1090m, 1078m, 979m, 964m, 916w, 837m, 769s, 760s, 731s, 697s, 648m, 584w, 510w, 493w, 472w.

*2-Phenyl-3-[N-(2-hydroxy-3-carboxybenzylidenamino)]-4(1H)-quinolinone* (**4**). Red crystalline product; yield: 589 mg (72%) after recrystallization from acetic acid, mp 272 °C; Anal. Calc. for C_23_H_16_N_2_O_4_ (M_r_ = 384.4): C, 71.9; H, 4.2; N, 7.3. Found: C, 71.4; H, 4.1; N, 7.0%; ESI− *m/z* (Int. %): 383 [M–H]^–^ (100), 767[2M–H]^–^ (10); ^1^H-NMR, DMSO*-d*_6_, *δ* (ppm), *J* (Hz): 6.70, 1H, *t*, 8.2, HC^23^, 7.47, 1H, *t*, 7.5, HC^6^, 7.67, 4H, *m*, HC^14,22,24^, HO^25'^, 7.74, 5H, *m*, HC^7,12,16,13,15^, 7.92, 1H, *d*, 7.5, HC^8^, 8.25, 1H, *d*, 8.5, HC^5^, 10.09, 1H, *s*, HC^18^, 12.66, *s*,HN^1^, 14.89, 1H, *s*,HO^20'^; ^13^C-NMR, DMSO*-d*_6_, *δ* (ppm): 115.68 (C7), 117.91 (C19), 118.00 (C21), 118.66 (C9), 119.43 (C23), 125.04 (C6), 125.56 (C11), 125.62 (C5), 129.59 (C13,15), 129.80 (C12,16), 131.34 (C14), 131.41 (C2), 133.07 (C24), 138.31 (C7), 138.59 (C3), 139.35 (C22), 147.36 (C10), 161.55 (C18), 168.01 (C20), 171.73 (C4), 172.14 (C25); FTIR (ν, cm^–1^): 3259br, 3216br, 3061br, 2997br, 2910br, 2796br, 2654br, 1684s, 1625s, 1617vs, 1602vs, 1562vs, 1515vs, 1469vs, 1433vs, 1396s, 1320s, 1222s, 1186s, 1150s, 1113m, 1066m, 1018m, 975m, 940w, 848w, 754s, 700s, 670m, 651w, 598w, 540w, 491w.

*2-Phenyl-3-[N-(2-hydroxy-3-allylbenzylidenamino)]-4(1H)-quinolinone* (**5**). Yellow crystalline product; yield: 680 mg (85%), mp 252 °C; Anal. Calc. for C_25_H_20_N_2_O_2_ (M_r_ = 380.4): C, 78.9; H, 5.3; N, 7.4. Found: C, 78.5; H, 5.3; N, 7.3%; ESI+ *m/z* (Int. %): 381 [M+H]^+^ (100), 761[2M+H]^+^ (20); ^1^H-NMR, DMSO*-d*_6_, *δ* (ppm), *J* (Hz): 3.16, 2H, *d*, 6.7, HC^27^, 4.96, 1H, *m*, HC^29a^, 4.99, 1H, *d*, 12.0, HC^29b^, 5.85, 1H, *m*, HC^28^, 6.82, 1H, *t*, 7.8, HC^23^, 7.10, 1H, d, 7.8, HC^22^, 7.29, 1H, *dd*, 7.8, 1.5, HC^24^, 7.40, 1H, *tt*, 7.2, 1.9, HC^6^, 7.59, 3H, *m*, HC^13-15^, 7.63, 2H, *m*, HC^12,16^, 7.71, 2H, *m*, HC^7,8^, 8.25, 1H, *d*, 8.1, HC^5^, 10.09, *s*, HC^18^, 12.23, *br*, HN^1^, 12.89, 1H, *s*, HO; ^13^C-NMR, DMSO*-d*_6_, *δ* (ppm): 33.32 (C27), 116.17 (C29), 118.89 (C8), 119.14 (C23), 119.81 (C19), 124.10 (C6), 124.35 (C9), 125.90 (C24), 126.15 (C11), 127.23 (C21), 128.99 (C13,15), 129.88 (C12,16), 130.28 (C14), 130.73 (C5), 132.30 (C7), 132.40 (C22), 134.07 (C2), 136.90 (C28), 138.60 (C3), 148.23 (C10), 158.14 (C20), 164.12 (C18), 172.86 (C4); FTIR (ν, cm^–1^): 3253br, 3057br, 2995br, 2975br, 2907br, 2778br, 1677w, 1626s, 1606vs, 1570vs, 1520s, 1503vs, 1474vs, 1446vs, 1430s, 1350s, 1279m, 1258m, 1233m, 1190s, 1157m, 1117m, 1074w, 990m, 936w, 903m, 842m, 754s, 743s, 698s, 675m, 655m, 582w, 513w, 488w.

*2-Phenyl-3-[N-(2-hydroxy-5-chlorobenzylidenamino)]-4(1H)-quinolinone·H_2_O* (**6**). Pale yellow crystalline product; yield: 736 mg (89%), mp 281 °C. Anal. Calc. for C_22_H_17_N_2_O_3_Cl (M_r_ = 392.8): C, 67.3; H, 4.4; N, 7.1. Found: C, 67.1; H, 4.3; N, 6.9%; ESI+ *m/z* (Int. %): 375 [M+H]^+^ (100), 751[2M+H]^+^ (25); ^1^H-NMR, DMSO-*d*_6_, *δ* (ppm), *J* (Hz): 6.71, 1H, *d*, 8.8, HC^21^, 7.23, 1H, *dd*, 8.8, 2.7, HC^22^, 7.40, 1H, *tt*, 7.0, 2.3, HC^6^, 7.50, 1H, *d*, 2.6, HC^24^, 7.58, 3H, *m*, HC^13-15^, 7.61, 2H, *m*, HC^12,16^, 7.70, 2H, *m*, HC^7,8^, 8.25, 1H, *d*, 8.2, HC^5^, 10.20, 1H, *s*, HC^18^, 12.32, 1H, *s*, HN^1^, 12.55, 1H, *s*, HO^20'^; ^13^C-NMR, DMSO-*d*_6_, δ (ppm): 118.62 (C8), 119.13 (C6), 121.84 (C19), 122.66 (C9), 123.77 (C23), 124.28 (C14), 125.89 (C5), 126.30 (C11), 129.05 (C13,15), 129.77 (C12,16), 130.37 (C21), 131.04 (C24), 131.74 (C22), 132.41 (C7), 134.00 (C2), 138.48 (C3), 149.37 (C10), 158.90 (C20), 161.40 (C18), 173.27 (C4); FTIR (ν, cm^–1^): 3251br, 3196br, 3088br, 3059br, 2974br, 2929br, 2765br, 1686m, 1623s, 1605vs, 1563vs, 1520s, 1497vs, 1470vs, 1445s, 1437s, 1385m, 1360m, 1344s, 1273s, 1233m, 1184s, 1164s, 1118m, 1089m, 1028w, 1001w, 985w, 966w, 940m, 876w, 817w, 750s, 699s, 676m, 650m, 582w, 537w, 505w, 487w, 453w.

*2-Phenyl-3-[N-(2-hydroxy-5-nitrobenzylidenamino)]-4(1H)-quinolinone* (**7**). Tan crystalline product; yield: 566 mg (69%), mp 292 °C; Anal. Calc. for C_22_H_15_N_3_O_4_ (M_r_ = 385.4): C, 68.6; H, 3.9; N, 10.9. Found: C, 68.2; H, 4.0; N, 10.5%; ESI+ *m/z* (Int. %): 386 [M+H]^+^ (85), 771[2M+H]^+^ (100); ^1^H-NMR, DMSO*-d*_6_, *δ* (ppm), *J* (Hz): 6.84, 1H, *d*, 9.2, HC^21^, 7.42, 1H, *m*, HC^6^, 7.61, 3H, *m*, HC^13–15^, 7.64, 2H, *m*, HC^12,16^, 7.71, 2H, *m*, HC^7,8^, 8.07, 1H, *dd*, 9.2, 2.7, HC^22^, 8.26, 1H, *d*, 8.2, HC^5^, 8.40, 1H, *d*, 2.7, HC^24^, 10.36, 1H, *s*, HC^18^, 12.41, 1H, *s*, HN^1^ , 13.94, 1H, *s*,HO^20'^; ^13^C-NMR, DMSO*-d*_6_, *δ* (ppm): 118.06 (C7), 119.21 (C6), 119.92 (C19), 122.86 (C9), 124.51 (C14), 125.88 (C5), 126.28 (C11), 127.57 (C21), 128.07 (C24), 129.16 (C13,15), 139.59 (C12,16), 130.59 (C22), 132.60 (C7), 133.66 (C2), 138.49 (C3), 139.60 (C23), 149.76 (C10), 160.64 (C18), 166.53 (C20), 173.08 (C4); FTIR (ν, cm^–1^): 3250br, 3199br, 3138br, 3057br, 2990br, 2936br, 2829br, 2766br, 1623s, 1604vs, 1565vs, 1528vs, 1495vs, 1470vs, 1448s, 1432s, 1387m, 1334vs, 1295vs, 1260s, 1231s, 1185s, 1166s, 1115m, 1093s, 1027m, 980m, 962m, 947m, 922m, 897m, 819s, 761m, 747s, 698s, 646m, 633m, 586w, 516w, 498m, 477w.

### 3.4. Single Crystal X‑ray Crystallography

X‑ray data of suitable single crystals of **1**, **3** and **7** were collected on an Xcalibur^TM^2 diffractometer (Oxford Diffraction Ltd., Oxford, UK) with Mo Kα (Monochromator Enhance, Oxford Diffraction Ltd.) and Sapphire2 CCD detector at 100 K (**1**), (**3**), and 120 K (**7**), respectively. Data collection and reduction were performed using CrysAlis software (Version 1.171.33.52, version 1.168). [18] Structures were solved by direct methods using SHELXS‑97 and refined on *F^2^* using the full‑matrix least‑squares procedure. [19] Non‑hydrogen atoms were refined anisotropically. All the hydrogen atoms were found in differential Fourier maps and their parameters were refined using a riding model with *U*_iso_(H) = 1.2*U*_eq_ (CH, CH_2_, NH, OH) or 1.5*U*_eq_ (CH_3_). The molecular graphics were drawn and additional structural parameters were interpreted using DIAMOND software [20] Non-routine aspects of the structure refinement are as follows: In compound (**3**), the PLATON-SQUEEZE procedure was employed to remove reflections of disordered methanol molecule [21,22].

### 3.5. Fluorescence Measurements

Solid state emission spectra were collected on an Infinite M200Pro instrument (Tecan, Männerdorf, Austria) using 96-well microplates. Fluorescence solution spectra were recorded on an AvaSpec HS1024x 122TE spectrometer (Apeldoorn, The Netherlands) using 0.5 cm cuvettes at room temperature. The fluorescence quantum yields of the compounds **3**–**7** were determined in 100 μM EtOH–H_2_O (95:5 v/v) solution by comparison with the fluorescence of quinine sulfate in 0.1 M sulfuric acid (φ = 0.54), taken as a reference fluorescence standard. Fluorescence quantum yields were calculated using the comparative method (Equation (1)):

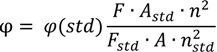
(1)
where φ(std) is the fluorescence quantum yield of standard F and F_std_ are the areas under the fluorescence emission curves of the measured compounds and standard respectively. A and A_std_ are the respective absorbance of the samples and standard at the excitation wavelengths. The terms *n* and *n_std_* are the refractive indices of solvents used for standard and samples [23].

### 3.6. In Vitro Cytotoxicity

*In vitro* cytotoxicity was studied by an MTT assay [MTT = 3-(4,5-dimethylthiazol-2-yl)-2,5-diphenyltetrazolium bromide] against osteosarcoma (HOS; ECACC No. 87070202) and breast adenocarcinoma (MCF7; ECACC No. 86012803) human cancer cell lines supplied from European Collection of Cell Cultures (ECACC) according to the literature [24]. The tested compounds **1**–**7**, H_2_*aqui* and cisplatin (used as a standard) were applied to the cells for 24 h at the concentrations up to 50 μM (0.01, 0.1, 1.0, 5.0, 25.0 and 50.0 μM). In parallel, the cells were treated with vehicle (DMF; 0.1% v/v; positive control to assess the minimal cell damage, *i.e*., 100% viability) and Triton X-100 (1% v/v; negative control to assess the maximal cell damage, *i.e*., 0% viability) to assess the minimal (*i.e*., positive control) and maximal (*i.e*., negative control) cell damage, respectively. The MTT assay was measured spectrophotometrically on a Tecan Schoeller Instruments LLC (540 nm). The cytotoxicity data from the respective cancer cell lines were acquired from three independent experiments (conducted in triplicate) using cells from different passages.

## 4. Conclusions

In this article we reported on the preparation and characterization of a series of seven new quinolinone-based Schiff base compounds **1**–**7**. All of the compounds were characterized by methods such as elemental analysis, FTIR and multinuclear NMR spectroscopy, and mass spectrometry. In the case of compounds **1**, **3** and **7**, the structures were determined by single crystal X-ray analysis. The compounds were studied for their fluorescence properties and cytotoxicity against HOS and MCF7 human cancer cell lines. The fluorescence experiments revealed that the compound **6** yielded the strongest intensity with the quantum yield of φ = 3.6% as compared to quinine sulfate as a standard. Unfortunately, no significant cytotoxicity (IC_50_ ˃ 50 µM) against HOS and MCF7 human cancer cell lines was found.

## Supplementary Materials

Supplementary materials can be accessed at: http://www.mdpi.com/1420-3049/19/9/13509/s1. Supplementary materials contain characterization of the starting compound 2-phenyl-3-amino-4(1*H*)-quinolinone, representative figures of 1D and 2D NMR spectra, electronic solid state and solution spectra of compounds **1**–**7**, deconvoluted fluorescence spectrum of **7** and a table of selected crystallographic non-covalent interactions for **1**, **3** and **7**. CCDC Nos. 1008032–1008034 contain the supplementary crystallographic data for **1**, **3**, and **7**, respectively. These data can be obtained free of charge via http://www.ccdc.cam.ac.uk/conts/retrieving.html, or from the Cambridge Crystallographic Data Centre, 12 Union Road, Cambridge CB2 1EZ, UK; fax: (44) 1223-336-033; or email: deposit@ccdc.cam.ac.uk.

## Supplementary Files

Supplementary File 1
