# Supplementary material for: Novel Schiff Bases Based on the Quinolinone Skeleton: Syntheses, X-ray Structures and Fluorescent Properties
Source: Molecules. 2014 Sep 1;19(9):13509–25. doi: 10.3390/molecules190913509 (PMC6270981; doi:10.3390/molecules190913509)
Supplement: Supplementary File 1 [file molecules-19-13509-s001.pdf]

Supplementary

Characterization of 2-Phenyl-3-amino-4(1*H*)-quinolinone

The compound was characterized by elemental analysis and ESI+ mass spectrometry. Anal. Calc. for $C_{15}H_{12}N_2O$ (M_r = 236.3): C, 76.3; H, 5.1; N, 11.9. Found: C, 76.4; H, 5.0; N, 11.3%. ESI+ *m/z* (Int. %): 237 $[M+H]^+$ (100), 276 $[2M+K]^+$ (10).

Figure S1. ^{13}C APT NMR spectrum of compound **3**.

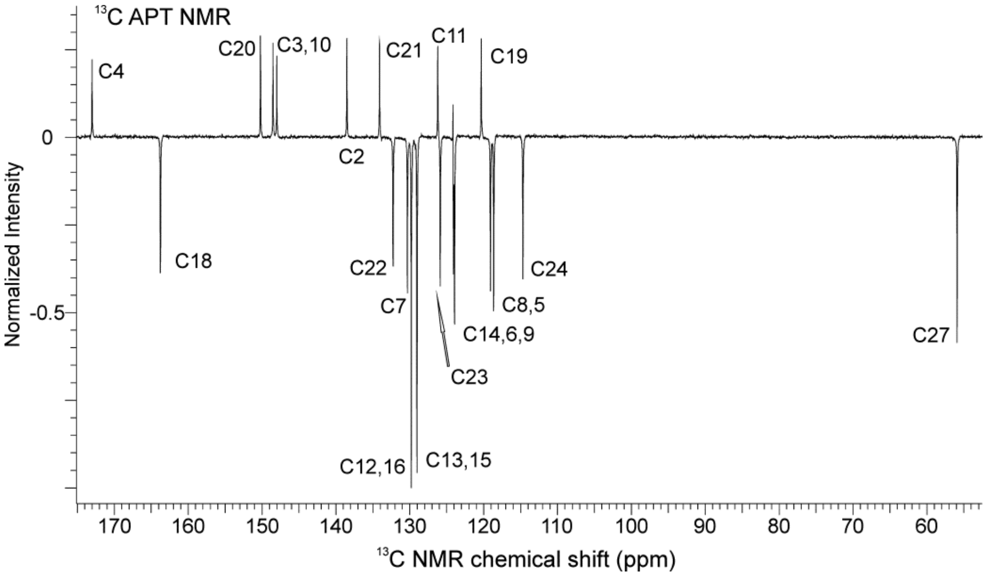

Figure S2. ^{1}H–^{13}C gs-HMQC NMR spectrum of compound **3**.

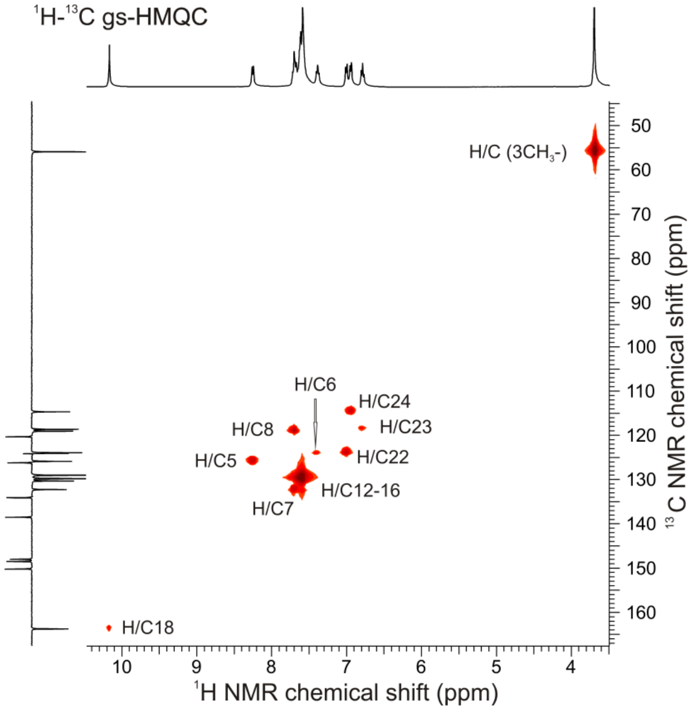

Figure S3. ESI− mass spectrum of **3** showing the molecular peak at 370 *m/z* and pseudo-molecular peak of a dimer at 739 *m/z*.

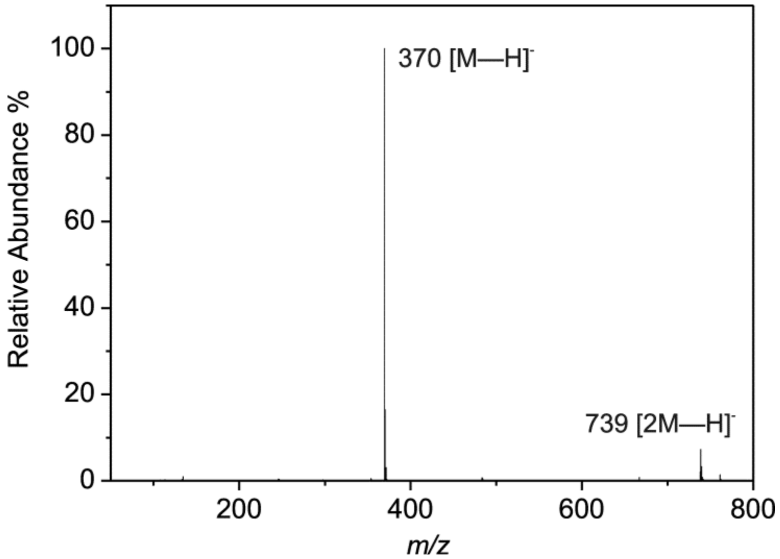

Figure S4. Electronic spectra of compounds **1**–**7**, 10 μM in EtOH–H_2O (95:5 v/v).

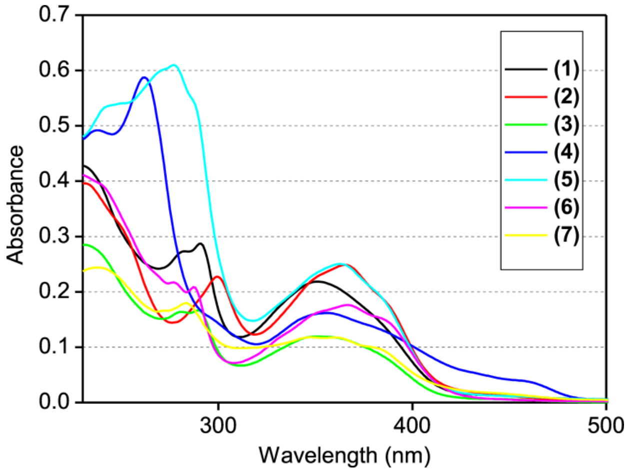

Figure S5. Electronic solid state spectra of compounds **1**–**7**.

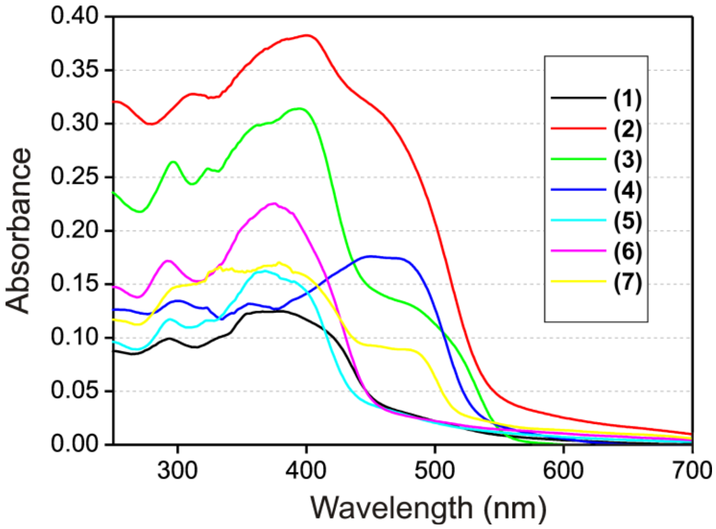

Figure S6. A deconvoluted fluorescence spectrum of compound **7**, showing two emission maxima at 521 nm and 575 nm.

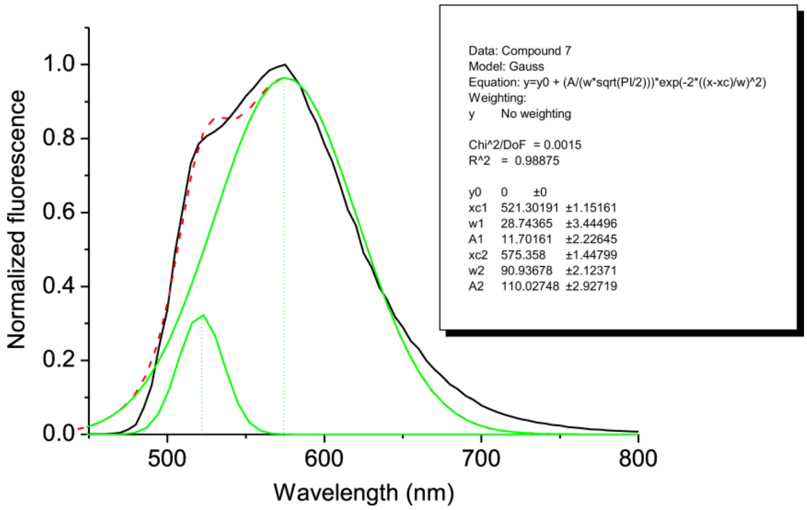

Table for compounds **1**, **3** and **7**.

Table S1. Selected non-covalent contacts (Å, °) for compounds **1**, **3** and **7**.

**D-H···A**	***d*(H···A)/Å**	***d*(D···A)/Å**	**∠ (DHA)/°**	**Symmetry codes**
		**1**		
O1···N1	1.862(1)	2.599(2)	145.74(9)	x,y,z
N2···O3	2.030(1)	2.885(2)	163.4(1)	1−x, −0.5+y, 0.5−z
N2···O1	2.482(1)	3.067(2)	124.43(9)	1−x, −0.5+y, 0.5−z
O3···O2s	1.739(2)	2.594(2)	159.0(1)	x, y, z
O2s···O2	1.89(2)	2.751(2)	161(2)	−x, −0.5+y, 0.5−z
O2s···O2	1.90(2)	2.711(2)	168(2)	x, 1.5−y, −0.5+z
		**3**		
O1···N1	1.825(1)	2.565(2)	146.09(9)	x, y, z
N2 ···O1	2.370(1)	3.198(2)	156.7(1)	1−x, 0.5+y, 0.5−z
N2···O3	2.188(1)	2.871(2)	134.1(1)	1−x, 0.5+y, 0.5−z
		**7**		
O1···N1	1.871(2)	2.615(3)	147.0(1)	x, y, z
N2···O2	1.950(2)	2.806(3)	164.1(1)	−0.25+x, 0.25−y, −0.25+z
